# Alternative Expression Pattern of MALT1-A20-NF-**κ**B in Patients with Rheumatoid Arthritis

**DOI:** 10.1155/2014/492872

**Published:** 2014-05-26

**Authors:** Xu Wang, Lihua Zhu, Ziwei Liao, Fan Zhang, Ling Xu, Yan Xu, Shaohua Chen, Lijian Yang, Yi Zhou, Yangqiu Li

**Affiliations:** ^1^Institute of Hematology, Jinan University, Guangzhou 510632, China; ^2^Key Laboratory for Regenerative Medicine of Ministry of Education, Jinan University, Guangzhou 510632, China; ^3^Department of Rheumatism and Immunology, First Hospital Affiliated, Jinan University, Guangzhou 510632, China; ^4^Guangdong Province Key Laboratory of Molecular Immunology and Antibody Engineering, Jinan University, Guangzhou 510632, China

## Abstract

Rheumatoid arthritis (RA) is an inflammatory autoimmune disorder; abnormal T cell immunity plays a critical role in the development of RA. Recently, A20 was identified as a key negative regulator for T cell activation and inflammatory signaling and may be involved in RA pathogenesis. In this study, we analyzed the expression level of A20, NF-*κ*B, and A20 regulatory factor mucosa-associated lymphoid tissue lymphoma translocation gene 1 (MALT1) in patients with RA. Real-time PCR was used to determine the expression level of MALT1, MALT-V1, A20, and NF-*κ*B genes in RA and healthy individuals (HI). Significantly lower A20 expression was found in RA patients compared with those in the healthy group, while NF-*κ*B overexpression could be detected in patients with RA. Moreover, the MALT1 and MALT1-V1 expression level was downregulated in RA patients. A positive correlation between MALT1 and A20 and MALT1-V1 and A20 was found in patients with RA, and a tendency towards a negative correlation was found between MALT1 and NF-*κ*B, MALT1-V1 and NF-*κ*B, and A20 and NF-*κ*B. In conclusion, we first characterized the alternative expression pattern of MALT1, A20, and NF-*κ*B in RA, which may be related to abnormal T cell activation.

## 1. Introduction

Rheumatoid arthritis (RA) is a common, chronic, systemic, and inflammatory autoimmune disorder that primarily affects the small diarthrodial joints of the hands and feet, affecting approximately 1% of the world's population [1–3]. The main characteristics of this disease are synovium hyperplasia, lymphocyte infiltration, and the abnormal proliferation of fibroblast-like synoviocytes (FLS) that can lead to the destruction of bone and cartilage and eventual disability [4]. Abnormal T cell immunity plays a critical role in the development of RA. Numerous factors that are involved in alternative T cell activation have been characterized, including the activation of inflammatory cells and expression of various cytokines. Inflammatory mediators, such as interleukin-6 (IL-6), interleukin-1 (IL-1), and tumor necrosis factor-*α* (TNF-*α*), are abundant in synovial tissues and fluid from patients with RA, and the overexpression of these cytokines promotes chronic inflammation and joint destruction. Many of the inflammatory mediators involved in the pathology of RA are regulated by nuclear factor kappa B (NF-*κ*B) transcription factors [5, 6]. Abnormal NF-*κ*B activation occurs during many pathological conditions including allergic and autoinflammatory diseases and malignancies [7].

Recently, A20, a negative regulator of NF-*κ*B, was identified as a key regulator for inflammation signaling and may be involved in RA pathogenesis [8]. A20 has been reported to be ubiquitin-editing enzyme with several functions. A20 is also known as tumor necrosis factor-*α*- (TNF-*α*-) induced protein 3 (TNFAIP3), which was first discovered in 1990 by Dixit and colleagues as a cytokine-induced gene in human umbilical vein endothelial cells [9, 10]. Subsequent studies demonstrated that A20 overexpression inhibits NF-*κ*B activation in response to different stimuli [11–13]. The cloning and characterization of the A20 promoter revealed two NF-*κ*B DNA binding elements, which are recognition sequences for NF-*κ*B transcription factors. It was also found that multiple NF-*κ*B activating stimuli induce A20 expression via NF-*κ*B sites in the A20 promoter [14]. Therefore, A20 has been demonstrated to downregulate its own expression, and it has been proposed that A20 participates in a negative feedback loop to attenuate TNF-*α*-induced inflammatory responses. A20 overexpression was subsequently demonstrated to block the NF-*κ*B activation mediated by TNF-*α*, IL-1, LPS, phorbol esters, and hydrogen peroxide in different cell types [11, 12, 15–18]. This inhibition is most likely due to the inhibition of NF-*κ*B activation in endothelial cells in response to proinflammatory stimuli, and an antiproliferative effect on smooth muscle cells has been observed upon A20 overexpression* in vitro*. All of these findings suggest that A20 attenuates the activity of proximal signaling complexes at proinflammatory receptors [19–21].

A20 is regulated by the CARMA1-Bcl-10-MALT1 (mucosa-associated lymphoid tissue lymphoma translocation gene 1) upstream signaling pathway complex, which bridges T cell antigen receptor (TCR) signaling with the canonical I*κ*B kinase (IKK)/NF-*κ*B pathway [20, 22–25]. TCR stimulation induces the recruitment of A20 and the Bcl-10 adaptor protein into the MALT1 complex, leading to MALT1-mediated A20 processing. Similarly, API2-MALT1 expression results in A20 cleavage. MALT1 cleaves A20 at arginine 439 and impairs its NF-*κ*B inhibitory function. Therefore, A20 was identified as a MALT1 substrate, emphasizing the importance of the MALT1 proteolytic activity in “fine-tuning” T cell antigen receptor signaling [26].

A20 dysfunction by deletion or mutation was identified in numerous lymphocytic malignancies [27]. Recently, polymorphisms in the A20 region were reported in autoimmune diseases such as systemic lupus erythematosus (SLE), rheumatoid arthritis (RA), Crohn's disease, and psoriasis. Single nucleotide polymorphisms in the A20 region, including rs13192841, rs2230926, and rs6922466, have been independently associated with increased susceptibility for SLE [28, 29], and this finding provides a critical link between A20 and the etiology of SLE. More recently, it was shown that A20 deficiency in myeloid cells triggers erosive polyarthritis, resembling RA in a myeloid-specific, A20-deficient mice model [30]. There are three strongly associated genetic variants, including rs6920220, rs6927127, and rs6933404, which result in an A20 functional decrease in RA [8].

The etiology of RA remains to be understood; however, A20 deficiency may be a pivotal regulator for inflammation in RA. To characterize the role of A20 in RA, we analyzed the expression level of A20, NF-*κ*B, and the A20 regulatory factor MALT1 in samples from Chinese patients with RA in this study.

## 2. Materials and Methods

### 2.1. Samples

This study included 16 patients with untreated RA (age: 26–71 years) and 20 healthy individuals (age: 22–70 years) who served as controls. The diagnosis of RA was based on the American College of Rheumatology criteria and expert opinion (1987 ACR criteria). All patients with RA were assessed for clinical disease activity by a trained rheumatologist using the disease activity score (DAS) [28]. The most recent erythrocyte sedimentation rate (ESR), C-reactive protein (CRP), and rheumatoid factor (RF) were collected (Table 1) [31].

All of the procedures were conducted according to the guidelines of the Medical Ethics Committee of the Health Bureau of Guangdong Province of China. Peripheral blood samples were collected by heparin anticoagulation, and peripheral blood mononuclear cells (PBMCs) were isolated using the Ficoll-Hypaque gradient centrifugation method. RNA extraction and cDNA synthesis were performed according to the manufacturer's instructions.

### 2.2. Quantitative Real-Time RT-PCR (qRT-PCR)

The sequences of the primers for MALT1, A20, and NF-*κ*B gene amplification are listed in Table 2. According to the structure of the MALT1 gene, there are two variants, that is, MALT1-V1 and MALT1-V2, and the latter contains a 33 bp deletion located between exons 6 and 8. To amplify the two MALT1 transcript variants, the MALT-V1-for and MALT-V1-rev primer pair was designed for MALT1-V1 amplification to cover the region that is missing in MALT1-V2, and the MALT1-for and MALT1-rev primer pair was designed to amplify the conserved region, which is contained by both variants.

The expression level of the A20, MALT1, MALT1-V1, NF-*κ*B, and *β*2-microglobulin (*β*2M) genes was determined by SYBR Green I real-time PCR. Briefly, PCR in a 20 *μ*L total volume was performed with approximately 1 *μ*L of cDNA, 0.5 *μ*M of each primer pair, 9 *μ*L of 2.5 × Real Master Mix (Tiangen Biotech (Beijing) Co., Ltd., Beijing, China), and 9 *μ*L of dH_2_O. After initial denaturation at 95°C for 15 minutes, 45 cycles of the following procedure were performed: 30 seconds at 95°C and 40 seconds at 60°C for the *β*2M, MALT1-V1, MALT1, A20, and NF-*κ*B genes. The plate was read immediately after the 60°C step using an MJ Research DNA Engine Opticon 2 PCR cycler (Bio-Rad, Hercules, CA, USA) [32]. The relative amount of the genes of interest and *β*2M reference gene was measured in two independent assays. The specific, amplified PCR products were analyzed by melting curve analysis. The data are presented as the relative expression of the genes of interest compared with the internal control gene as determined by the 2(^−ΔCT^) method [33]. In addition, to analyze the MALT1-V1 expression characteristics, we calculated the MALT1-V1 expression ratio as MALT1-V1/MALT1 × 100%.

### 2.3. Statistical Analysis

Two independent-samples Wilcoxon tests were performed to compare the median expression level for each gene between patients with RA and the control group. Pearson correlation and linear regression analyses were used to determine the association between different genes in different groups. A *P* < 0.05 was considered statistically significant [34].

## 3. Results and Discussions

**Figure 1:**
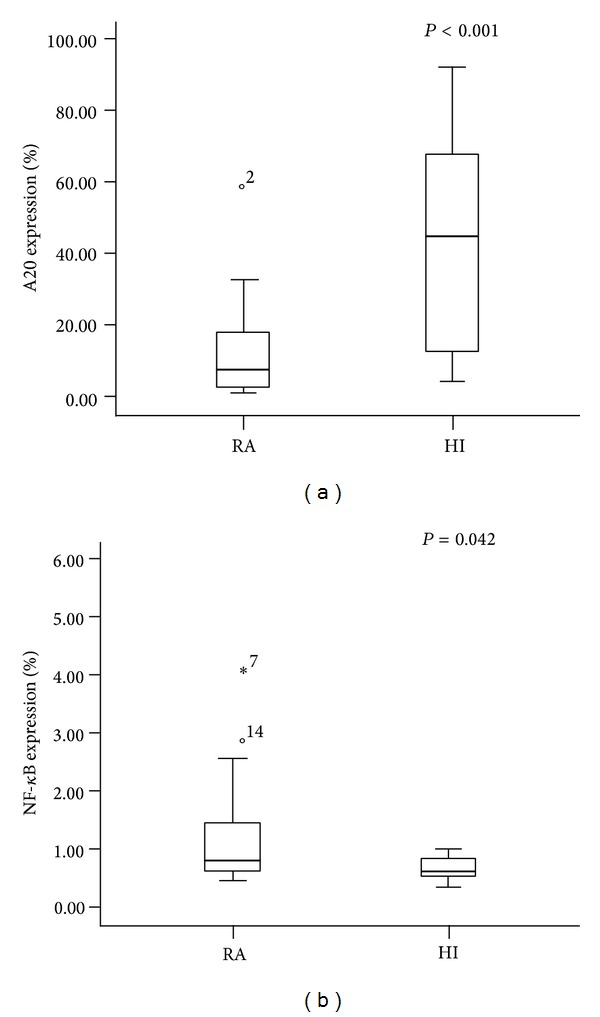
The expression level of A20 and NF-*κ*B in patients with RA and healthy individuals.

Abnormal T cell activation is a common feature of RA [35], and the upregulation of some positive regulating factors such as TNF-*α*, IL-6, and IL-2 was identified during the initiation of RA. In contrast, the downregulation of negative regulatory factors has the same effect on initiation of RA [16]. For example, T cell activation leads to the downregulation of A20 expression in mature thymocytes and peripheral T cells [9, 10]. Recently, abnormal A20 expression was described in patients with RA [8], and decreased A20 results in increased NF-*κ*B expression and enhanced inflammation [25]. In this study, we analyzed the expression of A20 in 16 patients with RA in the active phase, and a significantly lower expression of A20 (median: 6.530) was found compared with those in the healthy group (median: 44.614, *P* < 0.001) (Figure 1(a)). These results are similar to findings of different reports examining mouse models or patients with RA [8, 36]. A20-deficient mice develop severe multiorgan inflammation. Moreover, it is well accepted that the activation of NF-*κ*B-dependent gene expression plays a key role in the development of RA [30]; thus, decreased A20 expression may be a key reason for NF-*κ*B overexpression in RA. Our study also demonstrated that NF-*κ*B overexpression could be detected in patients with RA (median: 0.798) in comparison with healthy controls (median: 0.605, *P* = 0.042) (Figure 1(b)), indicating that decreased A20 resulting in NF-*κ*B overexpression is also a common feature for Chinese patients with RA.

**Figure 2:**
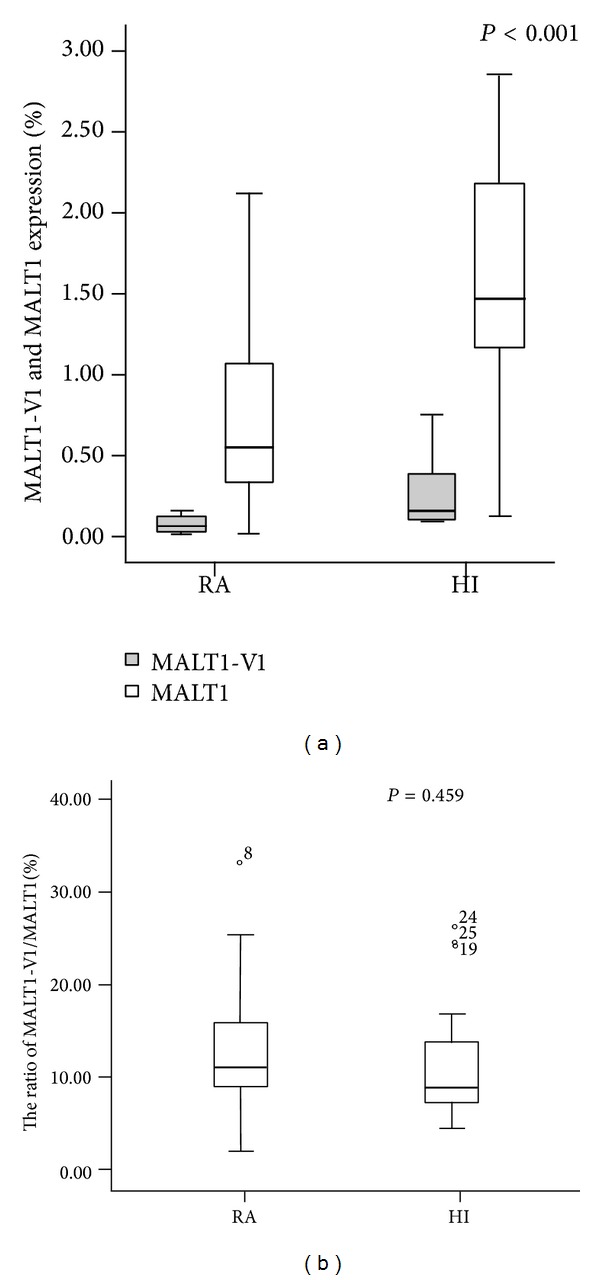
The expression level of MALT1-V1 and total MALT1 in patients with RA and healthy individuals.

Lower A20 expression is associated with polymorphisms in the A20 genomic locus [29]; however, whether there is any dysregulation in A20 by upstream pathway factors is unknown. MALT1 is an upstream A20 pathway factor that cleaves A20 at arginine 439 and impairs its NF-*κ*B inhibitory function. To characterize the relationship between MALT1 and A20 expression, we also examined the expression level of MALT1. Interestingly, the MALT1 expression level is downregulated in patients with RA (median: 0.541) compared with those in the healthy group (median: 1.638, *P* < 0.001) (Figure 2(a)). This result appears to be inconsistent with the lower A20 and higher NF-*κ*B expression level results because MALT1 is also a positive regulatory factor of NF-*κ*B. It is known that there are two MALT1 variants, MALT1-V1 and MATL1-V2, according to data in GenBank, and our previous study has found that these MALT1 variants could be identified by RT-PCR and sequencing (data not shown). In addition, we analyzed the expression level of the different variants, and similar results were found including the fact that a significantly lower MALT1-V1 expression level was detected in patients with RA (median: 0.062) compared with healthy controls (median: 0.140, *P* < 0.001) (Figure 2(b)).

Because we could not directly amplify MALT1-V2, which contains a 33 bp deletion, the expression level of MALT1-V2 could only be indirectly calculated by the relative expression of MALT1-V1/total MALT1 [37], and there are no significant differences in the ratio of MALT1-V1/total MALT1 between patients with RA and healthy controls (13.43 ± 7.98% versus 11.76 ± 6.66%), implying that the MALT1-V2 expression level was also downregulated in RA. Overall, either MALI1-V1 or MALT1-V2 was decreased in RA, unlike the finding in T cells from acute myeloid leukemia (AML), in which we found that the MALT1-V1 expression level was significantly higher in T cells from AML patients compared with healthy controls, while the MALT1-V2 expression level was downregulated [37]; this may indicate different expression pattern of these two MALT1 variants in RA. Little is known about the functional difference between the variants, and whether this is a feedback response from the expression pattern of A20 and NF-*κ*B in RA is unknown because the impact of A20 cleavage by MALT1 on its capacity to regulate NF-*κ*B has only been partially elucidated [24]. Further investigation is needed to characterize the upstream pathway regulators of A20 in addition to MALT1.

The role of MALT1 in the development of inflammation is largely unknown for RA and other autoimmune diseases, and only one study has reported that patients who had MALT-type lymphomas may also suffer from rheumatoid arthritis due to MALT1 dysfunction and continuous NF-*κ*B activation [38]. A study has reported that MALT1 rearrangement in gastric MALT lymphoma is frequently associated with Sjögren's syndrome [39]. More recently, Brüstle A and coworkers indicated that MALT1 is a central cell intrinsic factor that determines the experimental autoimmune encephalitogenic potential of inflammatory Th17 cells* in vivo* [40]. It appears that MALT1 may play a role in the development of autoimmune diseases, and it may interfere with specific T cell subsets. Thus, our finding of lower MALT1 expression may imply a loss of the control of T cell activation in some T cell subsets in RA, which remains an open question. Further studies are needed to investigate the pathways upstream of MALT1 and TCR-CD3 signaling in different T cell subsets in RA.

**Figure 3:**
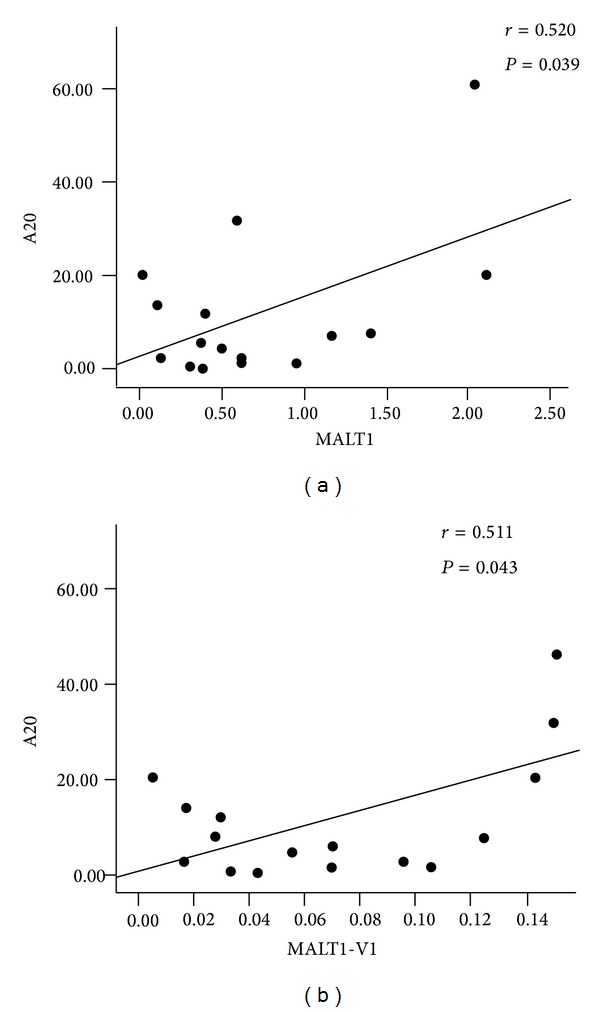
Correlation between the gene expression levels of MALT1 and A20 (a) and MALT1-V1 and A20 (b) in patients with RA.

In general, A20 is cleaved by MALT1; thus, the expression level of both genes should be negatively correlated with the expression pattern of A20 and MALT1 [24]. However, we found a positive correlation between MALT1 and A20 (*r* = 0.520, *P* = 0.039) (Figure 3(a)) and MALT1-V1 and A20 (*r* = 0.511, *P* = 0.043) (Figure 3(b)) in RA, and a tendency towards a negative correlation was found between MALT1 and NF-*κ*B (*r* = −0.098, *P* = 0.718), MALT1-V1 and NF-*κ*B (*r* = −0.204, *P* = 0.448), and A20 and NF-*κ*B (*r* = −0.264, *P* = 0.322), indicating that the MALT1-A20-NF-*κ*B expression pattern may be more complex in RA.

In conclusion, we characterized, for the first time, the alternative expression pattern of MALT1, A20, and NF-*κ*B in RA, which may be related to abnormal T cell activation. Lacking A20 and MALT1 dysfunction are common characteristics of Chinese patients with RA, and our results provide new inflammation targets to consider for RA treatment. Moreover, further investigation is needed to follow up on patients with different MALT1-A20-NF-*κ*B expression patterns and their association with cancer development.

## Figures and Tables

**Table 1:** Characteristics of RA samples.

Patient number	Gender	Age	Disease duration (mo)	RF (IU/mL)	ESR (mm/h)	CRP (mg/L)	DAS28 scores	CCP status
1	F	17	48	252.00	73	38.90	6.00	+
2	F	56	24	1940.00	111	7.40	6.78	+
3	F	51	2	17.50	77	37.70	7.09	+
4	F	43	60	74.30	37	5.34	5.53	+
5	F	62	72	38.30	69	17.00	6.88	+
6	F	32	120	19.90	85	9.06	7.51	ND
7	F	60	6	150.00	90	103.00	6.09	ND
8	F	26	9	31.00	32	1.84	4.31	+
9	F	53	6	368.00	89	31.90	7.41	−
10	F	54	9	9.19	64	33.25	7.39	−
11	F	45	12	102.00	82	68.98	6.26	+
12	F	53	240	65.30	76	27.08	7.91	+
13	F	71	12	58.90	110	71.35	6.79	+
14	F	63	12	153.00	32	4.84	5.23	+
15	F	33	84	299.00	42	18.70	5.61	+
16	F	60	6	10.10	41	0.57	6.63	−

Note: mo: months; F: female; +: positive; −: negative; ND: no detection.

**Table 2:** List of primer information.

Primer	Sequences	Accession number	PCR products
A20-for	5′-CTGGGACCATGGCACAACTC-3′	NM_006290	182 bp
A20-rev	5′-CGGAAGGTTCCATGGGATTC-3′		
MALT1-V1-for	5′-AAGCCCTATTCCTCACTACCAG-3′	NM_006785.2	195 bp
MALT1-V1-rev	5′-CACTCCACTGCCTCATCTGTTC-3′		
MALT1-for	5′-TCTTGGCTGGACAGTTTGTGA-3′	NM_006785.2	230 bp
MALT1-rev	5′-GCTCTCTGGGATGTCGCAA-3′		
NF-*κ*B-for	5′-CCACAAGACAGAAGCTGAAG-3′	NM_003998	149 bp
NF-*κ*B-rev	5′-AGATACTATCTGTAAGTGAACC-3′		
*β*2M-for	5′-TACACTGAATTCACCCCCAC-3′	J00105	145 bp
*β*2M-rev	5′-CATCCAATCCAAATGCGGCA-3′		
